# SENP3/FIS1-regulated PFC neural mitochondrial fragmentation underlies the mechanism of electroacupuncture attenuating depressive behavior in CUMS mice

**DOI:** 10.3389/fpsyt.2025.1645757

**Published:** 2026-01-02

**Authors:** Shaoda Lai, Xiaoke Qiu, Peilin Lin, Jiayi Wu, Feifei Li, Ningjing Lai, Xin Li, Ting Tu, Jiping Zhang, Yong Huang, Zhinan Zhang

**Affiliations:** 1 School of Traditional Chinese Medicine, Southern Medical University, Guangzhou, Guangdong, China; 2 Nanfang Hospital, Southern Medical University, Guangzhou, Guangdong, China; 3 Guangdong Provincial Key Laboratory of Chinese Medicine Pharmaceutics, Southern Medical University, Guangzhou, Guangdong, China; 4 Guangdong Basic Research Center of Excellence for Integrated Traditional and Western Medicine for Qingzhi Diseases, Guangzhou, Guangdong, China

**Keywords:** electroacupuncture, depression, prefrontal cortex, mitochondrial fragmentation, SENP3/FIS1

## Abstract

**Objective:**

Electroacupuncture (EA) is a common alternative treatment for depression, but its underlying mechanism remains unclear. Research suggests that its therapeutic effect may involve reducing SENP3/FIS1-regulated mitochondrial fragmentation, thereby mitigating neuronal damage in the prefrontal cortex. This study aimed to evaluate the efficacy of EA at Baihui (GV20) and Yintang (GV29) on SENP3/FIS1-regulated mitochondrial fragmentation in prefrontal cortex neurons of depressive animals.

**Methods:**

Twenty-eight 6-8-week-old male C57BL/6 mice were randomly divided into normal control, depression, and EA groups. Following depression modeling, the EA group received EA at GV20 and GV29. The effects of EA on neuronal mitochondrial fragmentation and the SENP3/FIS1 pathway were evaluated using transmission electron microscopy, western blotting, and immunofluorescence assays.

**Results:**

EA at GV20 and GV29 notably reduced depression-like behaviors in animals and exerted neuroprotective effects on prefrontal cortical neurons. It also inhibited FIS1-mediated mitochondrial fragmentation in the prefrontal cortex and enhanced SUMOylation. Further investigation of SENP3, the key regulatory enzyme for FIS1 SUMOylation, revealed that EA downregulated the SENP3-FIS1 interaction.

**Conclusions:**

These evidences suggested that the antidepressant effects of EA may involve modulation of mitochondrial fragmentation regulated by the SENP3/FIS1 pathway in prefrontal cortex neurons.

## 1 Introduction

Depression is a common mental disorder characterized by its high prevalence, recurrence, and detrimental impact. Globally, it affects approximately 264 million people of all ages, with a prevalence of about 5% among adults, and poses a significant risk to adolescents and the elderly (1). Depression ranks as the leading cause of disability worldwide and contributes heavily to the global burden of disease (2, 3). Although antidepressants such as paroxetine and citalopram are commonly prescribed, challenges remain due to their slow onset of action and potential side effects, including insomnia, sexual dysfunction, weight gain, nausea, diarrhea, and constipation (4, 5). Moreover, some antidepressants increase the risk of cerebrovascular disease (6).

Electroacupuncture (EA) is a commonly used alternative therapy for depression. Acupoints such as Baihui (GV20) and Yintang (GV29) are frequently used in the treatment of depression (7). Our previous research indicated that EA offers a faster onset and safer therapy for depression compared to conventional antidepressants, and that EA has similar efficacy to manual acupuncture (acupuncture without electric currents) (8). Although growing evidence supports the therapeutic benefits of EA in depression, its precise molecular mechanisms remain to be elucidated (9).

Research has revealed prefrontal cortex (PFC) atrophy is one of the pathological features in both patients with depression and animal models, primarily resulting from neuronal destruction and atrophy (10, 11). Mitochondria are crucial organelles for cellular energy production and play important roles in various cellular pathways, including apoptosis, calcium homeostasis, inflammation, and immunity (12). Mitochondrial fragmentation, characterized by the breakdown of mitochondria into small and dispersed structures, is an indicator of mitochondrial dynamic imbalance (13). This process is mediated by dynamin-related protein 1 (DRP1) and its receptors, mitochondrial fission 1 protein (FIS1) and mitochondrial fission factor (MFF) (14). Emerging evidence suggests that EA may improve mitochondrial function in neurons, specifically by addressing mitochondrial division and fragmentation damage (15).

SUMO (small ubiquitin-like modifier) modification is a post-translational modification that regulates the function of covalently attached target substrates by binding to specific lysine residues, thereby affecting the cell cycle, DNA repair, and signal transduction (16). SUMO modification is involved in the regulation of mitochondrial dynamics (fission and fusion) and central nervous system (CNS) diseases, such as Parkinson’s disease and cerebral ischemia-reperfusion injury (17, 18). SUMO modification has been implicated in both the pathogenesis of depression and the regulation of mitochondrial fragmentation (19). SUMO-specific protease 3 (SENP3), which specifically regulates SUMO modification of FIS1, decreases FIS1 SUMOylation, thereby enhancing FIS1-regulated mitochondrial fragmentation (20). Our previous study indicated that EA at GV20 and GV29 modulates SUMO-modification-related pathways in depressive states (21). Based on this evidence, we postulate that EA may attenuate prefrontal neuronal damage by reducing SENP3/FIS1-regulated mitochondrial fragmentation and protecting prefrontal neurons.

In this study, we investigated the mechanisms underlying EA intervention at GV20 and GV29 in animal models of chronic unpredictable mild stress (CUMS)-induced depression. We also examined the effects of EA on neuronal mitochondrial fragmentation and the SENP3/FIS1 pathway, thereby providing new insights into the mechanisms of action of EA in depression treatment.

## 2 Materials and methods

### 2.1 Animals

Twenty-eight male C57BL/6 mice (6–8 weeks old), purchased from Southern Medical University Experimental Animal Center (Guangdong, China; license No. SYXK (Yue) 2016-0167), were housed in an SPF facility (temperature 24 ± 2°C, humidity 50%-60%, light/dark cycle 12h/12h) with free access to water and food. After seven days of adaptation, the mice were randomly divided into a CUMS model group (CM, n=20) and a control group (NC, n=8), using a random number generator of SPSS software (IBM, USA), and the CUMS modeling process began. The Southern Medical University Experimental Animal Ethics Committee approved the study protocol (No. L2017178), in accordance with the United States National Institutes of Health Guide for the Care and Use of Laboratory Animals (NIH Publication No. 85-23, revised 1986).

### 2.2 CUMS model

The CUMS model is commonly used to simulate the onset and symptoms of depressive conditions (22). All animals in the CUMS model group (CM, n=20) underwent a 28-day CUMS procedure (23). The mice were randomly exposed to stressors, including noise (24 h), water deprivation (24 h), food deprivation (24 h), inversion light/dark cycle (12/12 h), strobe illumination (12 h), restraint (12 h), and level shaking (5 min). These stressors were administered randomly, with 1–2 stressors per day, and the same stressor was not used on consecutive days to prevent animals from predicting the occurrence of stimulation. After the modeling, if sucrose preference rate in sucrose preference test (SPT) is higher than 80%, the animal is considered failed the modeling and were excluded (24). Approximately 80% of the CUMS model mice were successfully induced, and the four mice that failed to develop depressive behavior were excluded. Ultimately, 16 CUMS model animals were successfully established.

### 2.3 Interventions

Sixteen successfully modeled mice were randomly assigned to either the CUMS group (n=8, sham electroacupuncture) or the EA group (n=8, EA interventions) by a random number generator of SPSS software (IBM, USA), with 10 mice in each group. The NC group (n=8) continued with free access to water and food.

#### 2.3.1 EA

**Figure 1:**
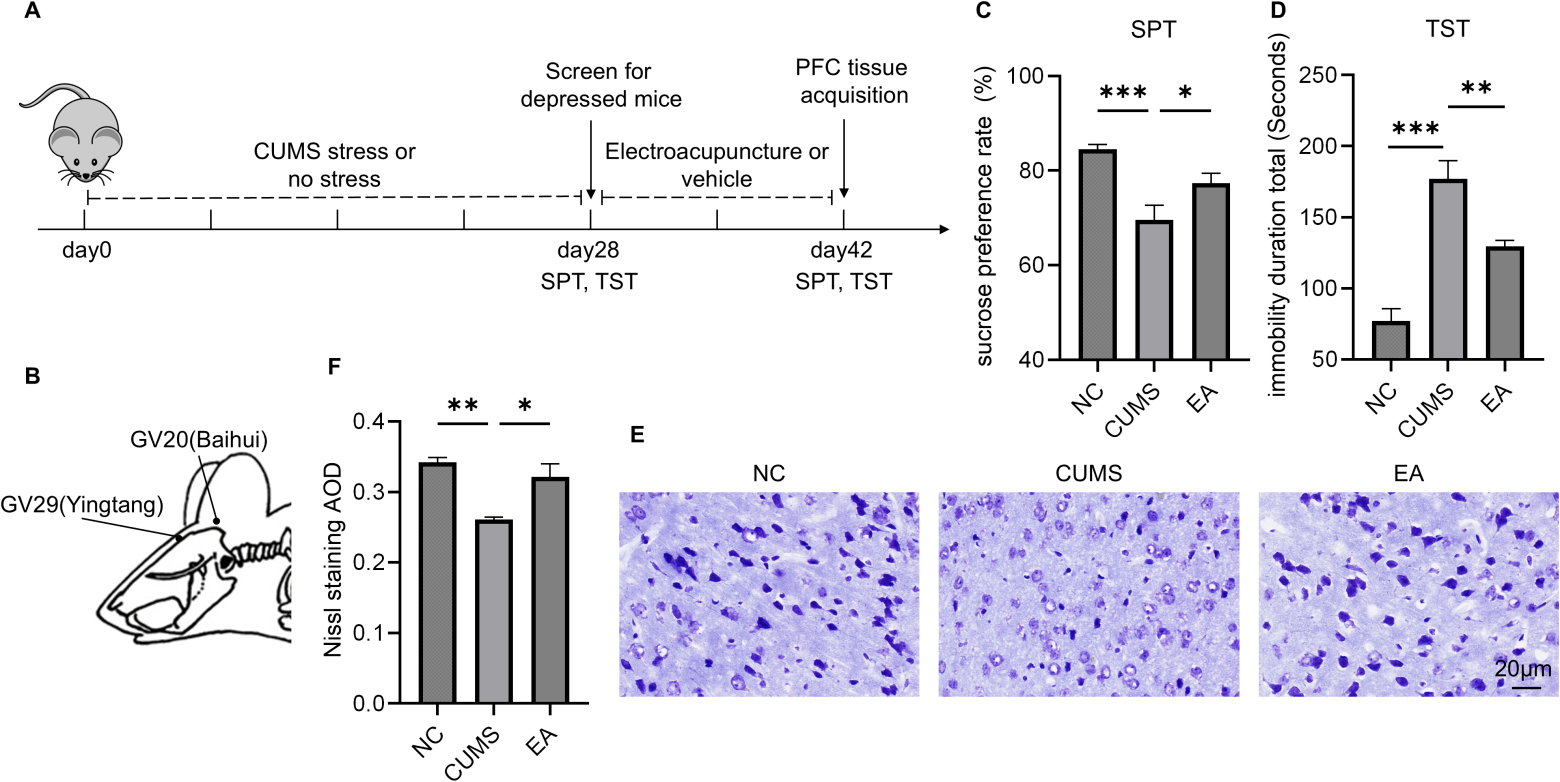
EA ameliorates depressive behaviors and PFC neural lesions in depressive mice. **(A)** Study design; **(B)** The location of GV20 and GV29 in mice; **(C)** Sucrose preference rate in SPT; **(D)** Immobility duration in TST; **(E)** Representative graphs of PFC Nissl staining, Bar = 20μm; **(F)** AOD of positive PFC neuron in Nissl staining; *P<0.05, **P<0.01, ***P<0.001.

After the animals were mildly immobilized in a centrifuge tube with medical adhesive tape, exposing the head for EA, Baihui (GV20, at the center of the head, midpoint of the auricular apices) and Yintang (GV29, at the midpoint between the brow bones) were chosen for EA intervention (**Figure 1B**) (25). EA was performed at the two acupoints using disposable sterile needles (0.22 × 5 mm, Suzhou Acupuncture and Moxibustion Supplies Co., Ltd., Jiangsu, China; depth: 2–3 mm, angle to the skin: 15°; direction: toward the tip of the nose), which were secured with tape. Subsequently, the two needles were connected to the electrodes of the HANS EA device (LH-202H, Lianchuang Science and Technology (Group) Nanjing Jisheng Medical Technology Co., Ltd., Jiangsu, China) using electrode wires, with GV20 connected to the positive pole and GV29 to the negative pole; sparse and dense waves were applied (1 mA, 2/15 Hz, 5V). EA was performed for 30 min once daily for 14 days.

#### 2.3.2 Sham EA

After mild fixation, GV20 and GV29 were selected, and blunt-tipped disposable sterile needles were used. The needles were fixed on the body surface with tape but did not penetrate it. They were connected to the electrodes of HANS EA instrument, with the same waveform, current, and frequency parameters as those of EA, but with pre-destroyed wires. Sham EA was performed for 30 min once daily for 14 days.

### 2.4 Behavioral tests

The SPT and tail suspension test (TST) were used to examine depressive behaviors in the animals at the end of the intervention. The animals were euthanized the day after the behavioral experiments (**Figure 1A**).

#### 2.4.1 Sucrose preference test

The SPT was used to assess anhedonic behavior, which is the core (24). Briefly, before the formal SPT, the animals were acclimated to a 1% (w/v) sucrose solution in a quiet room. Animals were housed individually. Two bottles of sucrose solution were placed in each cage for 24 h, after which one bottle was replaced with pure water for 24 h. Following acclimation, the animals were deprived of water and food for 12 h.

During the 24-hour SPT, each animal had access to two bottles: one containing 1% (w/v) sucrose solution and the other containing pure water. The positions of the bottles were switched every 12 h to prevent place preference, and each bottle was weighed before and after testing. The sucrose preference rate was calculated using the following formula: sucrose solution consumption/(sucrose solution consumption + water consumption) × 100%. Body weights were measured at the end of the intervention period.

#### 2.4.2 Tail suspension test

The TST was used to assess desperate behavior (26). In a quiet room, animals were suspended from the top of an opaque apparatus using tape affixed approximately 1 cm from the tail tip. To avoid experimenter influence, the apparatus was isolated from the experimenter during the test. Prior to each test, the apparatus was sanitized with alcohol to remove residual odors from the previous subject. Animals were suspended for 6 min, and the last 4 min of immobility were recorded analysis. Immobility duration was analyzed using SMART3.0 software (Panlab, Spain).

### 2.5 Tissue acquisition

After intraperitoneal injection with sodium pentobarbital (60 mg/kg), the bilateral ventromedial prefrontal cortex (vmPFC) tissues were isolated. Mice were randomly assigned to different tissue acquisition groups. For 3 animals/group, the left brain, used for Nissl staining and immunofluorescence staining, was fixed with 4% paraformaldehyde for 48 h and routinely embedded in optimal cutting temperature (OCT) compound. The right brain (3 animals/groups), intended for transmission electron microscopy (TEM), was immediately cut into 1 mm^3^ pieces and preserved in 2.5% glutaraldehyde (G5882, Sigma-Aldrich, Massachusetts, USA) until TEM processing. For samples used in immunoblotting (3 animals/groups), PFC tissues were washed at 4°C in phosphate-buffered saline (PBS) to remove excess blood and stored at -80°C until needed. All samples were labeled with initials and numbers to minimize bias.

### 2.6 Nissl staining

Tissues were routinely embedded in OCT and sectioned in a freezing microtome (CM1850, Leica, Hessen, Germany) to produce 30 μm-thick slides. After Nissl staining, dehydration, transparency, and sealing, neurons were observed under a microscope at 400× magnification. Six randomly selected, non-overlapping fields of view were photographed for each animal. The average optical density (AOD) values of the captured images was analyzed using ImageJ software.

### 2.7 Transmission electron microscopy

Tissues were immersed in 1% osmium tetroxide for 1 h and then rinsed with PBS three times for 15 min each. Next, the samples were soaked in a gradient of acetone (50%, 70%, and 90% each for 15 min; 100%, 3x, for 15 min). The tissues were soaked in a mixture of acetone and Spurr’s resin (acetone:Spurr’s resin = 1:1 for 1 h; acetone: Spurr’s resin = 1:2 for 2 h), then immediately placed in 100% Spurr’s resin overnight and embedded in Spurr’s resin in coffin molds, which were cured for 8 h in an oven at 70°C. PFC tissues were sliced at 60 nm using an ultramicrotome and counterstained with saturated aqueous uranyl acetate and Reynolds lead citrate (3x for 5 min). Sections were examined using TEM (H-7500, Hitachi, Tokyo, Japan) and photographed at 3,000g and 8,000graphededsred3A8 Four neurons were randomly selected per animal and photographed using ImageJ to analyze the average area and diameter of mitochondria.

### 2.8 Western blotting

Tissues were homogenized in radioimmunoprecipitation assay (RIPA) lysis buffer at 4°C for 30 min. Following centrifugation at 12,000 rpm for 15 min, protein content was determined using the bicinchoninic acid (BCA) assay. Samples containing 20 μg of protein were loaded onto a 10% SDS-PAGE gel and transferred onto PVDF membranes. After blocking with BSA/TBST at room temperature for 3 h, the membranes were incubated with primary antibodies, including DRP1 (1:1500, 12957-1-Ap, Proteintech, Chicago, USA), MFF (1:4000, 66527-1-Ig, Proteintech), FIS1 (1:1000, SAB2702049, Sigma-Aldrich), SUMO2/3 (1:1500, 11251-1-Ap, Proteintech), SENP3 (1:1000, 17659-1-Ap, Proteintech), and GAPDH (1:3000, HRP-60004, Proteintech), overnight at 4°C. Secondary antibodies were incubated at room temperature for 1 h (1:3000, anti-rabbit SA00001-2, Proteintech; 1:3000, anti-mouse SA00001-1, Proteintech). WB images were captured using enhanced chemiluminescence (ECL) and a gel imaging system (UVP Company, Upland, California, USA). Band intensities were analyzed using ImageJ software.

### 2.9 Immunofluorescence

Tissues were embedded in OCT and sectioned in a freezing microtome to produce 30 μm-thick slides. Slices were rinsed with PBS, and incubated with FIS1 (1:400, 66635-1-Ig, Proteintech) and SENP3 (1:400, 17659-1-Ap, Proteintech) antibodies overnight at 4°C. After rinsing with PBS, the sections were incubated with secondary antibodies (1:300, green, RS3211, Immunoway, California, USA; 1:300, red, RS3608, Immunoway) for 2 h at room temperature and blocked with a blocking solution containing DAPI. Images of FIS1 and SENP3 were obtained using a laser confocal microscope (63x oil lens, LSM 800, ZEISS, Baden-Württemberg, Germany).

### 2.10 Statistical analysis

Required sample sizes were estimated based on other similar experiments (24, 27). For normally distributed data, results are expressed as mean ± SEM. Statistical significance was assessed using one-way ANOVA followed by Dunnett’s *post hoc* multiple comparisons test. For non-normally distributed data, values are presented as medians with interquartile ranges. Nonparametric analyses, including Kruskal-Wallis one-way ANOVA, were employed, followed by Dunn’s *post hoc* correction. All analyses were conducted using GraphPad Prism 10 (GraphPad, California, USA), and all statistical tests were two-sided. Statistical significance was set at P < 0.05.

## 3 Results

### 3.1 EA mitigates depressive behaviors and ameliorates PFC neural lesions in depressive mice

After 14 days of EA intervention, the SPT results showed that the sucrose preference rate was significantly higher in the EA group compared with the CUMS group (P < 0.05, **Figure 1C**). Moreover, the TST showed a significant reduction in immobility time in the EA group compared with the CUMS group (P < 0.01, **Figure 1D**). These findings suggested that EA at GV20 and GV29 alleviated the primary symptoms of depression in CUMS mice, particularly anhedonia and desperate behaviors.

Given the PFC’s crucial role in the pathogenesis of depression, Nissl staining of the PFC was performed to assess neuronal pathological changes. Staining results demonstrated a significant increase in AOD in the EA group compared with the CUMS group (P < 0.05, **Figures 1E, F**). Additionally, neurons in the EA group appeared more orderly arranged. These observations suggested that EA ameliorated neuronal lesions in the PFC of depressed mice.

### 3.2 EA reduces mitochondrial FIS1-regulated PFC neural mitochondrial fragmentation in depressive mice

**Figure 2:**
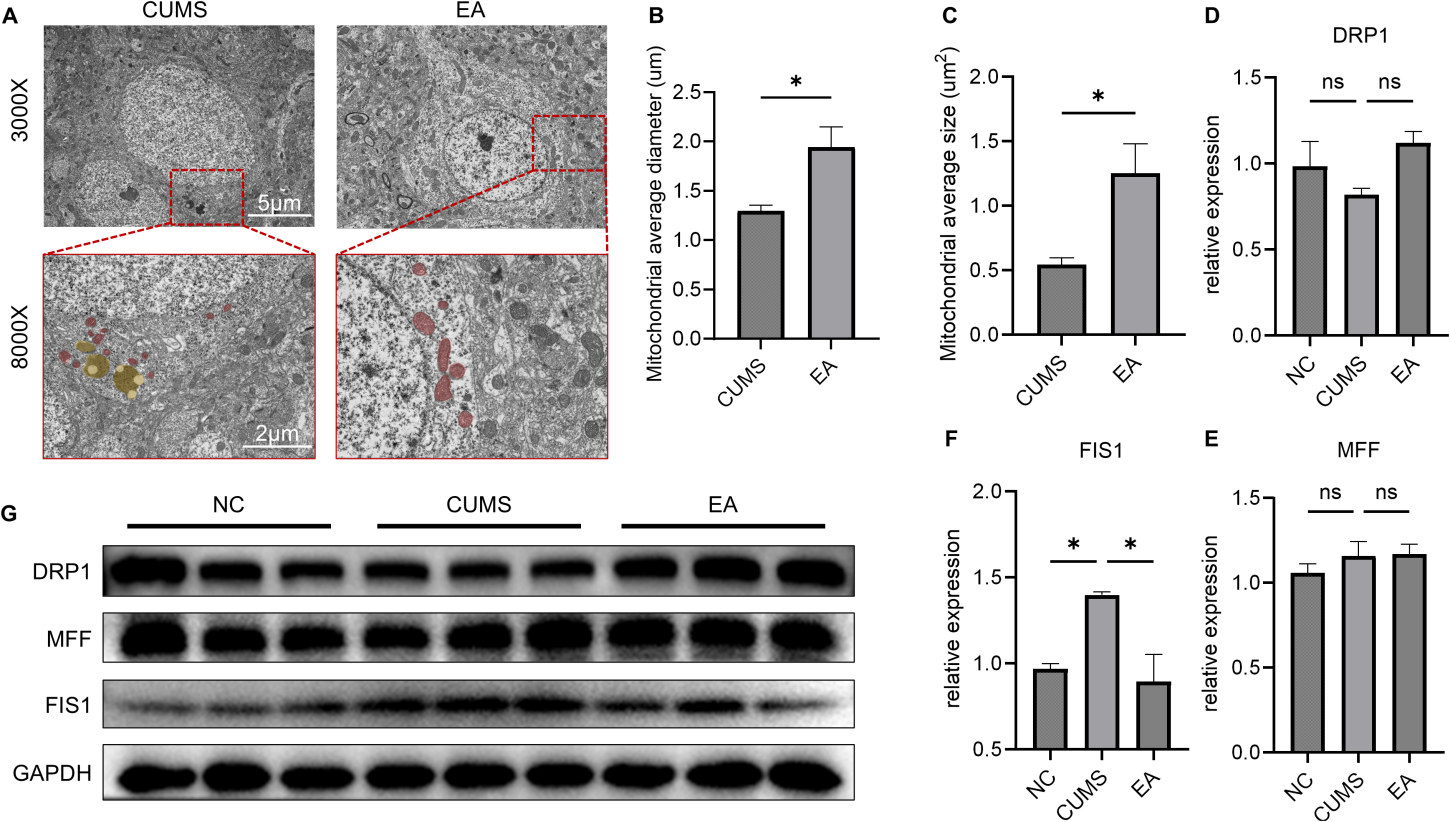
EA reduces FIS1-regulated PFC neural mitochondrial fragmentation in depressive mice. **(A)** Representative TEM images of PFC neural mitochondria ultrastructure, Bar = 5μm, 2μm (enlarged), red: neural mitochondria, yellow: autolysosome; **(B)** Average mitochondrial diameter in PFC neurons in TEM; **(C)** Average mitochondrial size in PFC neurons in TEM; **(D)** Histogram of the relative expression of DRP1; **(E)** Histogram of the relative expression of MFF; **(F)** Histogram of the relative expression of FIS1; **(G)** Western blotting images of PFC DRP1, MFF, and FIS1; *P<0.05, ns, no significance.

TEM results revealed pronounced mitochondrial fragmentation in PFC neurons of the CUMS group, characterized by swelling, cristae disorganization, and reduced mitochondrial diameter. Conversely, PFC neurons in the EA group exhibited normal mitochondrial morphology, intact cristae, and significantly longer diameters and larger regions compared with the CUMS group (P < 0.05, P < 0.05). Furthermore, autophagy lysosomes in PFC neurons were significantly reduced in the EA group compared to the CUMS group (**Figures 2A–C**). These findings indicated that EA alleviated depression in part by decreasing mitochondrial fragmentation in PFC neurons.

Although mitochondrial fragmentation is typically mediated by DRP1-regulated mitochondrial division, WB did not show a significant difference in DRP1 levels in the PFC between the EA and CUMS groups (P > 0.05; **Figures 2D, G**). However, further examination of mitochondrial DRP1 ligands MFF and FIS1 revealed that while MFF levels were not significantly different between the groups (P > 0.05; **Figures 2E, G**), FIS1 expression was significantly elevated in the EA group (P < 0.05; **Figures 2D, G**). These results suggested that EA might mitigate FIS1-induced mitochondrial fragmentation in depressive PFC.

### 3.3 EA reduces mitochondrial fragmentation via the SENP3/FIS1 pathway

**Figure 3:**
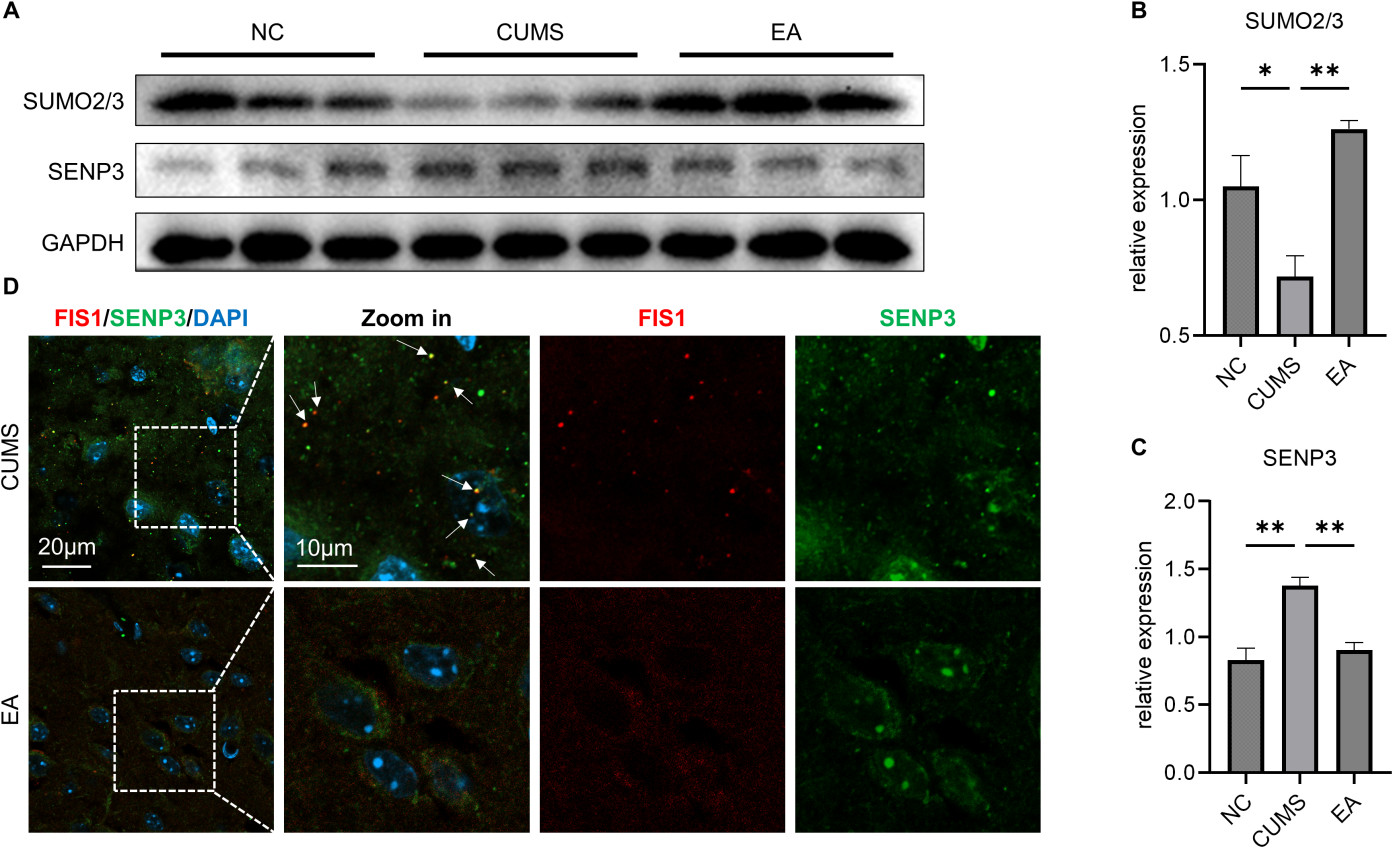
EA reduces mitochondrial fragmentation via the SENP3/FIS1 pathway. **(A)** Western blotting images of PFC SUMO2/3, SENP3; **(B)** Histogram of the relative expression of SUMO2/3; **(C)** Histogram of the relative expression of SENP3; **(D)** Representative graphs of PFC FIS1 and SENP3 co-localization in IF. *P<0.05, **P<0.01.

SUMO is a post-translational modification of FIS1 and is also involved in the pathogenesis of depression (19). Further analysis of SUMO modification in the PFC also showed that EA led to a significant increase in overall SUMO levels (P < 0.01, **Figures 3A, B**), indicating that EA’s efficacy is associated with enhanced SUMO modification within the PFC. Previous research has demonstrated that FIS1 activity is modulated by sentrin-specific protease 3 (SENP3), a SUMO-modified FIS1-specific hydrolase that deSUMOylates FIS1, thus augmenting its pro-fission activity (20). Our analysis revealed that EA significantly reduced SENP3 levels in the PFC of depressed animals (P < 0.01; **Figures 3A, C**). Furthermore, IF co-staining of FIS1 and SENP3 indicated that EA significantly decreased their co-localization (**Figure 3D**). These findings suggested that EA suppressed FIS1 and mitochondrial fragmentation, possibly by reducing SENP3-regulated deSUMOylation of FIS1, which enhancing the SUMOylation of FIS1 in depressed PFC.

## 4 Discussion

In this study, we investigated the efficacy of EA at GV20 and GV29 for depressive mice, with one mechanism of action being the alleviation of neuronal damage in the PFC. The results showed that EA ameliorated depressive-like behaviors. EA has also been reported to improve post-stroke depression and is safer than antidepressants (28). Additionally, EA at GV20 and GV29 can regulate extracellular ATP levels in the PFC, alleviating depression-like behavior in rats (29). These findings are consistent with those of this study. Therefore, reduction of neuronal damage in the PFC is one of the mechanisms underlying EA’s antidepressant effects.

Further research also indicated that EA significantly decreased FIS1-regulated mitochondrial fragmentation in PFC neurons. Mitochondrial fragmentation injury is primarily regulated by DRP1 and its receptor proteins (30). Generally, cytosolic Drp1 is recruited to the mitochondrial outer membrane by its receptors, such as MFF and FIS1, where they self-assemble into a ring-like structure and, through GTP hydrolysis, cleaves the mitochondrial membrane (31). In our study, we observed decreased mitochondrial fragmentation; however, DRP1 protein levels did not change significantly. Rather, we found that EA reduced the expression of one of its receptors, FIS1.

Mitochondrial fragmentation is caused by mitochondrial fission, which involves the division of a mitochondrion into two daughter mitochondria (32). Interestingly, a specific inhibitor of MFF-DRP1 binding was shown to worsen oxidative stress in septic cardiomyopathy, while a specific inhibitor of FIS1-DRP1 binding alleviated morbidity (33). Recent research has shown that MFF and FIS1 underlie the different fates of daughter mitochondria, in which normal mitochondrial division leads to new mitochondrial genesis is regulated by MFF, whereas abnormal mitochondrial division leading to mitochondrial fragmentation and mitophagy is regulated by FIS1 (34). Our results showed that FIS1 expression was downregulated following EA treatment. We also observed that autophagic lysosomes were reduced in the PFC neurons of EA-treated mice (**Figure 2A**). Therefore, it is hypothesized that EA alleviates mitochondrial fragmentation by suppressing FIS1-regulated mitochondrial fragmentation in PFC neurons, thereby reducing neuron lesions in PFC.

Posttranslational modification (PTM) plays important roles in mitochondrial dynamics (13). SUMOylation is one of the recently identified PTMs of FIS1 (35). SUMOylated FIS1 exhibits a diminished affinity for binding to mitochondria, resulting in reduced mitochondrial division (36). Our experiments showed that EA intervention significantly increased SUMO levels in the PFC, indicating that the effectiveness of EA is associated with increased SUMO modification in the PFC. Additionally, SENP3 can specifically reduce SUMO-modified FIS1, thereby enhancing FIS1 activity (37). We also found that EA decreased SENP3 levels and co-localization of SENP3 and FIS1. Recent research found that SENP3 is upregulated in the hippocampus of depressed animals and that elevated SENP3 impairs CREB-BDNF signaling, thereby causing hippocampal lesions (38). Therefore, these findings suggested that EA might mitigate PFC mitochondrial fragmentation via the SENP3/FIS1 pathway.

Nevertheless, this study has some limitations. The current investigation did not sufficiently explore the SENP3/FIS1 pathway. More research is needed to further examine the interaction between SENP3 and FIS1, including the use of SENP3 inhibitors to confirm the results. Furthermore, our results suggested that the mechanism of EA may also be linked to mitophagy, which warrants further investigation.

## 5 Conclusions

**Figure 4:**
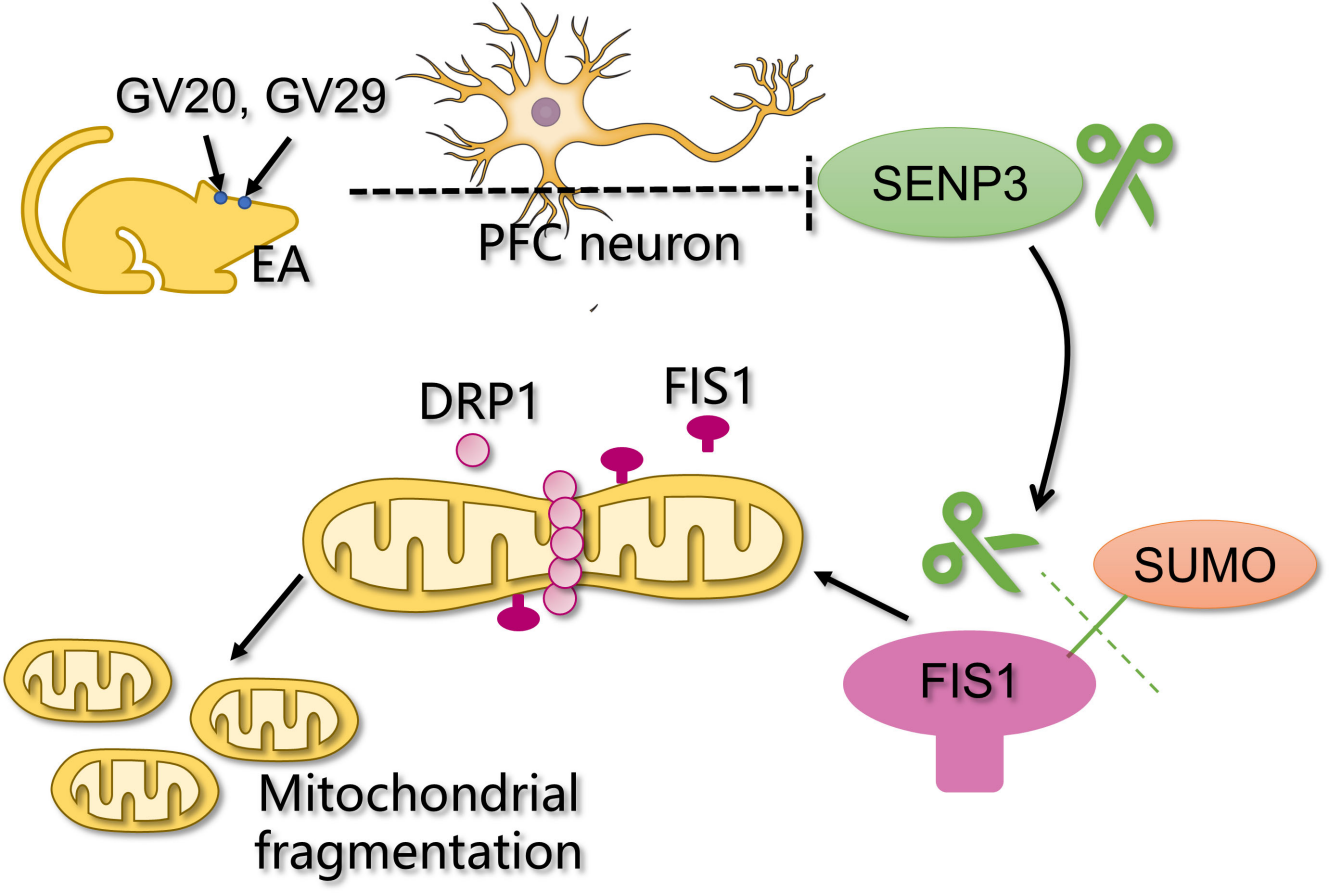
Schematic illustration of how EA alleviates depressive behaviors by mitigating PFC mitochondrial fragmentation via the SENP3/FIS1 pathway.

These findings suggested that the antidepressant effect of EA is likely related to the downregulation of SENP3 in PFC neurons, thereby increasing SUMO modification of FIS1, which decreases FIS1-mediated mitochondrial fragmentation injury and protects PFC neurons (**Figure 4**).

## Data Availability

The raw data supporting the conclusions of this article will be made available by the authors, without undue reservation.
